# Effect of Elevated Temperature on Compressive Strength and Physical Properties of Neem Seed Husk Ash Concrete

**DOI:** 10.3390/ma13051198

**Published:** 2020-03-06

**Authors:** Kizito Patrick Mwilongo, Revocatus Lazaro Machunda, Yusufu Abeid Chande Jande

**Affiliations:** 1 Department of Materials and Energy Science and Engineering, the Nelson Mandela African Institution of Science and Technology, P.O. Box 447, Arusha 23311, Tanzania; mwilongok@nm-aist.ac.tz; 2 Water Infrastructure and Sustainable Energy Futures, the Nelson Mandela African Institution of Science and Technology, Nelson Mandela Rd, P.O. Box 9124, Nelson Mandela, Arusha 23311, Tanzania; revocatus.machunda@nm-aist.ac.tz; 3 Department of Civil Engineering and the Built Environment, Saint Augustine University of Tanzania, P.O. Box 307, Mwanza 33114, Tanzania; 4 Department of Water and Environmental Science and Engineering, the Nelson Mandela African Institution of Science and Technology, P.O. Box 447, Arusha 23311, Tanzania

**Keywords:** neem seed husk ash, concrete, elevated temperature, spalling, mass loss, compressive strength

## Abstract

High temperature rise mostly caused by a fire outbreak is currently becoming a threat that endangers concrete’s structural performance for buildings and the safety of occupants. The behavior of concrete after fire subjection has been of much interest for the structural materials design purposes. This study investigated the physical properties and the compressive strength of M25 concrete incorporating Neem Seed Husk Ash (NSHA), exposed to and through targeted different levels of temperature (200 °C to 800 °C) for a period of three hours in an electric furnace. The NSHA was produced by calcining neem seed husks at 800 °C for six hours and then sieved through the 125 μm sieve. Different amounts of NSHA were investigated while considering the plain concrete as the control sample. 150 concrete cubes of 150 mm sizes were cast and properly cured for 7 and 28 days. The experimental results show that the compressive strength of the 5% NSHA concrete exposed to temperatures up to 400 °C is 21.3% and 23.8% better than the normal concrete at 7 and 28 curing days, respectively. Surface cracks and spalling are noticeable at 600 °C and 800 °C for all samples considered in this study.

## 1. Introduction

Concrete is a composite building material comprised of coarse and fine aggregates glued together with cement paste that results in the desired structure upon hardening. It is a material of choice for applications that require high-temperature-resistant materials. However, concrete structures can only serve their intended purpose, provided that the threshold point is not exceeded upon exposing them to elevated temperatures, like those in case of fire accidents [1].

The current development of infrastructures due to urbanization has led to about 11 billion tons of concrete being consumed annually, and is likely to exceed 18 billion tons by the year 2050 [2]. This implies that its raw materials, more particularly cement, are highly consumed. It is reported that in every ton of ordinary Portland cement (OPC) manufactured, approximately 900 kg of CO_2_ is also produced, contributing to about 7% of the entire CO_2_ emissions worldwide [3]. 

Hence, the issues of materials sustainability and environmental pollution accelerated by the concrete industry need to be addressed. Many researchers of our century have been looking for eco-friendly materials that can partially or totally replace cement in the mix with the aim of handling the raised concern on depletion of raw materials, atmospheric pollution, global warming and waste disposal [4]. However, such replacement should result in improved concrete properties in terms of strength and durability. 

Currently, pozzolans from agro-wastes and industrial wastes have been of much interest in the field of building materials as they contribute to blended cement concrete with superior properties parallel to the reduction of the aforementioned environmental problems [5]. With the growing interest in looking for eco-friendly building materials, researchers found a need for assessing the potential use of ash from agricultural wastes. Several investigations have been conducted to examine the properties of blended concrete at normal and elevated temperatures utilizing rice husk ash [6,7,8], palm oil fuel ash [9,10] and sugar cane bagasse ash [11,12] as admixtures in concrete production. The blended concrete was observed to perform better than normal concrete due to the presence of amorphous silica in the aforementioned synthetic pozzolanic materials [4,13]. Therefore, more materials with good properties can also be studied to save the purpose. For example, the increasing world’s demand for fuel has led more researchers to look for biodiesel from neem seeds which also produce a large number of wastes [14]. These wastes have a potential application as eco-friendly building materials, since neem seed husk ash (NSHA) is a good pozzolan and can partially replace cement in normal strength concrete [15,16,17,18]. However, materials used in concrete structures must meet certified fire-resisting constraints as stipulated by the assorted standards subject to the intended structural purpose. 

Meanwhile, there are complications on the fire endurance ability of traditional concrete by itself due to dissimilar thermal characteristics of the components forming the composite material, porosity and moisture content [5]. Generally, the OPC products are considered as good fire resisting structural materials which can still perform suitably even after a long time of exposure [19]. According to Khoury [20], with a careful selection of materials, the compressive strength of conventional concrete can be retained without considerable loss up to about a 550–600 °C temperature rise, although for exposures exceeding 200 °C, concrete’s internal stresses are high enough to cause cracking and notable damages which eventually lead to decreased compressive strength. It is therefore essential to investigate the properties of blended concrete to have a clear knowledge of the emerging materials before they are incorporated in concrete production. Since many factors like age of concrete, the inclusion of pozzolanic materials and the type of aggregates used all together influence the strength and macro properties of concrete at elevated temperatures; it is, therefore, important to examine the residual compressive strength for concrete under such exposures for structural safety [21]. A good number of available studies concentrated on assessing the influence of exposure time, the methods of cooling and loading behaviors on the mechanical properties of concrete and incorporating the processed pozzolanic materials from agro wastes like rice husk ash [22,23], palm oil fuel ash [5], bamboo ash [24] and sugar cane bagasse ash [12] subjected to elevated temperatures. Unlike previous studies, this study intends to investigate the residual compressive strength and physical response of blended cement concrete containing NSHA as pozzolanic materials after exposure to elevated temperatures up to 800 °C. A comparison is then made on residual strength and physical properties of NSHA concrete with the properties of the conventional concrete.

## 2. Materials and Methods

### 2.1. Materials

#### 2.1.1. Binder

In the present study, the OPC 42.5 N grade conforming to Tanzanian standard TZS727: 2002 in accordance with BS 12:1996 13 and Neem Seed Husk Ash (NSHA) were used. Their chemical composition and surface area will be presented and discussed under the results section.

**Figure 1:**
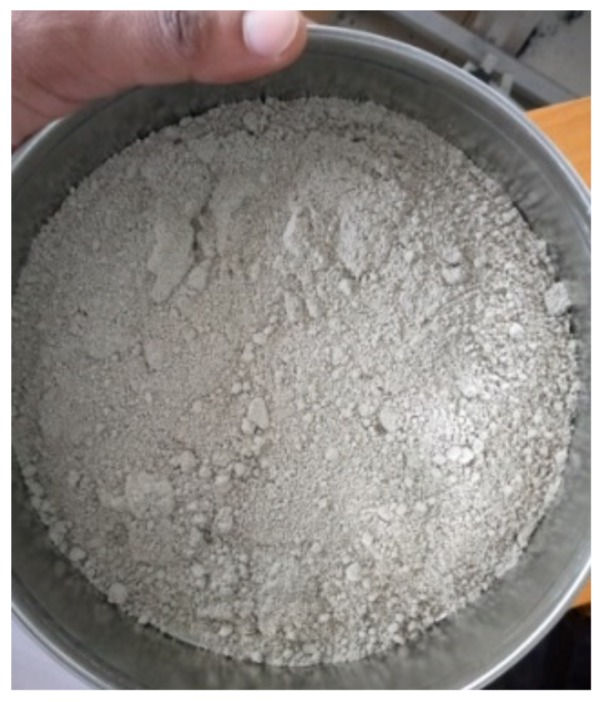
The NSHA used for this study.

The OPC was acquired from local suppliers in Arusha city, Tanzania. The NSHA was obtained by burning the neem seed husk in an electric furnace at 10 °C/min heating rate to a temperature of 800 °C for six hours followed by grinding and sieving through 125 μm sieve as visually presented in Figure 1. 

**Figure 2:**
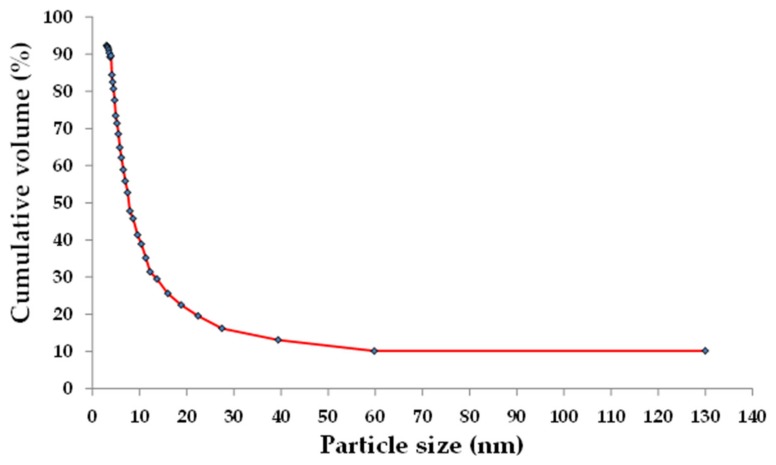
Particle size distribution of Neem Seed Husk Ash (NSHA).

The neem seed husks were locally collected from Singida, the central region of Tanzania. The moisture content was measured to be 1.8%, and they were oven-dried at 110 °C for six hours then ground to small size before calcining. X-ray fluorescence (XRF) used was to determine the chemical composition of NSHA, while the surface area was calculated with the Brunauer-Emmett-Teller (BET) method from nitrogen adsorption-desorption. The heating regimes were adopted from the previous study which assessed the pozzolanic activity of palm oil waste ash in response to calcining temperatures and retention time [25]. The particle size distribution of the NSHA is also illustrated in Figure 2. The NSHA passing through 45 μm sieve was considered for water absorption determination [26].

#### 2.1.2. Aggregates

The properties of the used aggregates were determined, guided by the standards. The BS EN 933-1:2012 [27] was used to confirm the particle size requirement for aggregates. ASTM C127 [28] and ASTM C128 [29] were used to determine the specific gravity and water absorption of coarse and fine aggregates respectively. 

**Figure 3:**
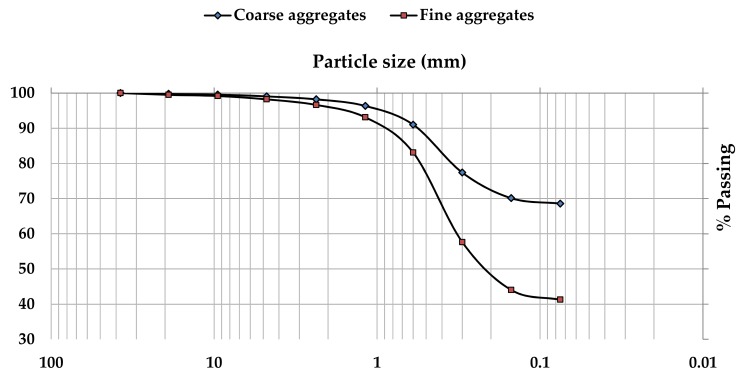
The particle size distribution for the used aggregates.

In this research work, natural river sand with fineness modulus of 2.6 was used as fine aggregates and crushed granite aggregates with a nominal maximum size of 20 mm were used as coarse aggregates. The particle size distribution and some of the measured properties of fine and coarse aggregates are presented in Figure 3 and Table 1, respectively. 

#### 2.1.3. Water

Portable tap water was utilized for mixing.

### 2.2. Mix Proportioning

Concrete was prepared by partially substituting cement by NSHA. The proportioning and the mixing of concrete ingredients were done as per BS 1881-125:1986 [30] for all concrete series. Five series of test specimen were considered in this research; one series of specimens of normal strength concrete (with 0% NSHA) and four series of specimens of cement-NSHA concrete with replacements of cement by 3%, 5%, 7%, and 10% NSHA as presented in Table 2. The targeted concrete grade was M25.

### 2.3. Specimen Preparation

The concrete blends were prepared in an electric mixer. The weighed fine and coarse aggregates were put into the concrete mixer and moisturized in advance before adding water to their saturation. Afterward, water was added with the addition of cement together with the NSHA and thoroughly mixed for three minutes. Standard cubes of size 150 mm were used. A total of 150 cubes were cast from which three cubes from each series were considered for each exposure condition. After casting, specimens were allowed to set under room temperature of 25 ± 3 °C. After 24 h all the concrete specimens were demolded and then cured by submerging in water under identical environmental conditions. At the targeted ages of 7 and 28 days, cubes were taken out of the water and exposed for 24 h to naturally dry under laboratory environmental conditions.

### 2.4. Heating and Cooling Regimes 

The prepared concrete samples were fired in a controlled electric furnace to different levels of temperature (200 °C, 400 °C, 600 °C and 800 °C) at 10 °C/min, a methodology similar to an earlier study [31], for a period of 3 h in each target temperature and then cooled to room temperature in the furnace to avoid cracking due to temperature gradient. 

### 2.5. Testing Program 

The control samples were weighed and tested with no heat application. All specimens other than the control specimens were weighed before exposing them to elevated temperatures. After cooling, samples were then weighed again and observed for any spalling that might have occurred during the heating process. The recorded weights were then used to determine overall mass loss at each temperature similar to previous studies [21]. Their residual compressive strength was measured using the Compressive Strength Testing Machine with maximum load capacity 3000 kN with a loading scale of 5 kN per division. The compression load was applied at the rate of 100 kN/min. The crushing loads were obtained by loading the samples to failure in accordance with BS EN 12390-3 [32]. The reported ultimate compressive strengths were calculated as the average of the three individual cubes in each series considered. A comparison was finally made on the properties of heated samples with reference to the control (unheated) samples.

## 3. Results and Discussion

### 3.1. Characterization of NSHA

The chemical composition of NSHA and the OPC were analyzed using X-ray Fluorescence (XRF). From the results presented in Table 3, the NSHA is having silica, aluminum oxide, iron oxide and calcium oxide, containing 45.2%, 22.6%, 3.4% and 2.6%, respectively. For fly ash, most of the compounds are silica and alumina. The NSHA used in this investigation was considered as pozzolanic material suitable for concrete, and can be classified as fly ash Class F, since the sum of silica, alumina and iron oxide exceeds 70% [33]. The NSHA had a larger surface area than the cement used; this fastened the reaction rate leading to enhanced early strength development.

The reactivity of fly ash, when employed as a partial substitution of cement in concrete mixes, depends to a large extent on chemical composition and the particle size distribution. The fraction of ashes passing the 45 μm sieve is suitable for use in concrete production as the reactivity of fly ash increases with its fineness [26]. Based on the particle size distribution of the NSHA presented in Figure 2, a large portion (80%) of the used NSHA has less than 45 μm particle size. The improved strength is highly expected as a result of the reactivity of the NSHA when it partially substitutes OPC in concrete.

### 3.2. Physical Characteristics with Temperature Rise

The evaluation of impairment to the concrete structures exposed to high temperatures was thoroughly done to observe the changes occurring to the surface of concrete specimens. Visual observation of the color changes, as well as of the spalling and cracks on the surface of the concrete is considered to be the first step in assessing the damages of fired concrete. Visual observation of fired concrete which was considered as the first step assessment discovered a similar pattern of cracks and pits on the surfaces of both the 7 and 28 days cured concrete samples. The physical characteristics of concrete at various temperatures up to 800 °C are presented in Table 4. For all ratios of concrete, the surfaces showed no significant change when subjected to temperatures up to 400 °C, while having the same appearance and color. At a temperature of 600 °C, cracking and spalling were observed and they became widely noticeable at 800 °C. The spalling behavior is associated with the generated pore pressures and growth of cracks, which in turn are affected by the rate of heating and the composition of the heterogeneous concrete. A similar observation was observed in previous work by Owaid et al. [21]. This suggests that the maximum safe temperature of 400 °C can be attained when NSHA admixtures are incorporated in concrete, though the behavior of concrete at temperatures between 400 °C and 600 °C might be considered for future works to be sure of the threshold temperature. 

### 3.3. Spalling and Mass Loss

**Figure 4:**
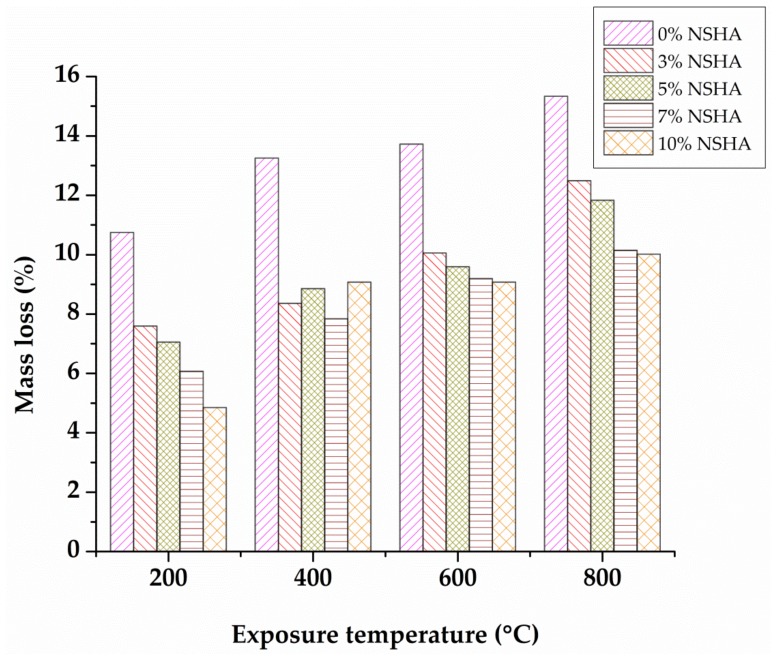
Mass loss versus temperature for OPC and NSHA concrete cured for 7 days.

The cube samples were respectively weighed before and after exposure to elevated temperatures for the purpose of evaluating the mass loss. Figure 4 displays the concrete mass loss in relation to the initial weight of the samples of OPC and cement-NSHA blended concrete properly cured for 7 days with increasing exposure temperature. It is clearly seen that non-NSHA concrete samples studied revealed a gradual increase in mass loss from 10.7% to 15.3% for the temperature increasing from 200 to 800 °C. The NSHA concrete samples showed a small mass loss as compared to the control mix (concrete samples with no NSHA) ranging from 4.8% to 10.0% for the temperature increasing from 200 to 800 °C. The experimental findings also indicate a decrease in a mass loss upon increasing the NSHA materials. Such a decrease in mass loss may be attributed by the dense matrices of the NSHA concrete, as compared to the mix which does not contain NSHA, as the mass loss is caused by air voids associated with the loss of free and bound water from the paste. Similar findings were previously reported [21,34]. Greater mass losses were observed for all batches considered at a temperature of 800 °C, which may be attributed to the evaporation of total absorbed and adsorbed water from concrete as it was also observed by Ramesh et al. [35].

**Figure 5:**
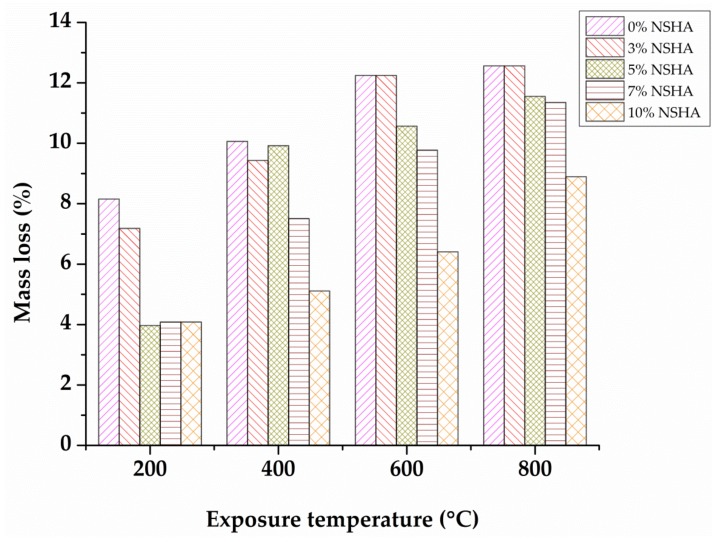
Mass loss versus temperature for OPC and NSHA concrete cured for 28 days.

The mass losses for the 28 days cured concrete samples are shown in Figure 5. It can be seen that there is a slight similarity in the trend of loss of weight with the increasing temperature as it was observed for the 7 cured samples. In this case, mass loss is gradually increased from 8.2% to 12.6% for the OPC concrete samples for the temperature increasing from 200 to 800 °C, respectively. The NSHA concrete samples experienced a mass loss increasing gradually from 4.0% to 8.9% for the temperature increasing from 200 to 800 °C, respectively. Increasing the exposure temperature directly increases the mass loss due to releasing of free water and the bound water-related to Ca(OH)_2_ decomposition and the other hydrated cement products developed during the hydration process. A previous study by Janotka and Nürnbergerová [34] observed similar results. The mass loss at temperatures above 400 °C was associated with disintegration, leaving some holes on the surface due to spalling. The results show that mass loss is higher for the early age concrete samples than for the mature concrete samples. For all samples, higher rates of mass loss were observed at temperatures extending from 200 to 400 °C, followed by a slight increase in the mass loss at 600 and 800 °C. The findings of the present study concur with observations by Fares et al. [36], who testified that nearly 70% of the free and bound water in the concrete evaporates at 300 °C.

### 3.4. Residual Compressive Strength 

The compressive strength for the cooled concrete samples after thermal treatment was determined. The residual strength for the 7 and 28 days cured concrete samples subjected to different elevated temperatures is presented in Table 5. The experimental results show a slight decrease in compressive strength for temperature rising up to 200 °C; followed by an increase in strength as the temperature rises to 400 °C before a noticeable drop in strength at temperatures of 600 °C and 800 °C for all samples containing 0%, 3%, 5%, 7% and 10% NSHA, as validated in prior studies [19,37,38]. 

**Figure 6:**
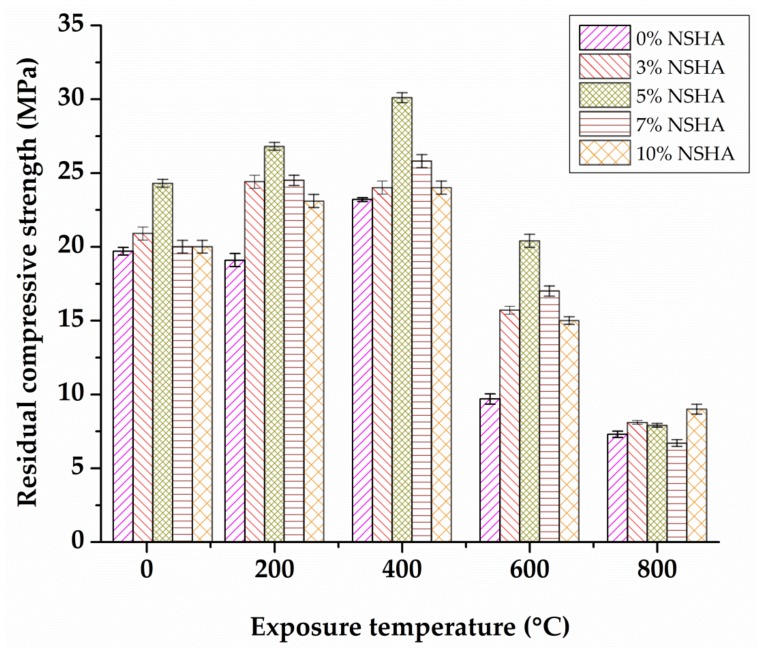
Residual compressive strength versus temperature for the 7 days cured concrete.

Figure 6 illustrates the relationship between the compressive strength of assorted cement pastes after 7 days of curing and temperature for which the concrete samples were exposed; the error bars are the standard deviations of the test results. For all batches of concrete, samples considered were observed to maintain their structural integrity for the temperatures ranging from 200–400 °C, while the sample with the optimal replacement of 5% NSHA still performed better up to 600 °C. 

In the prior investigation, the compressive strength increased at 250 °C and had higher residual strength at 450 °C and 650 °C [39].

**Figure 7:**
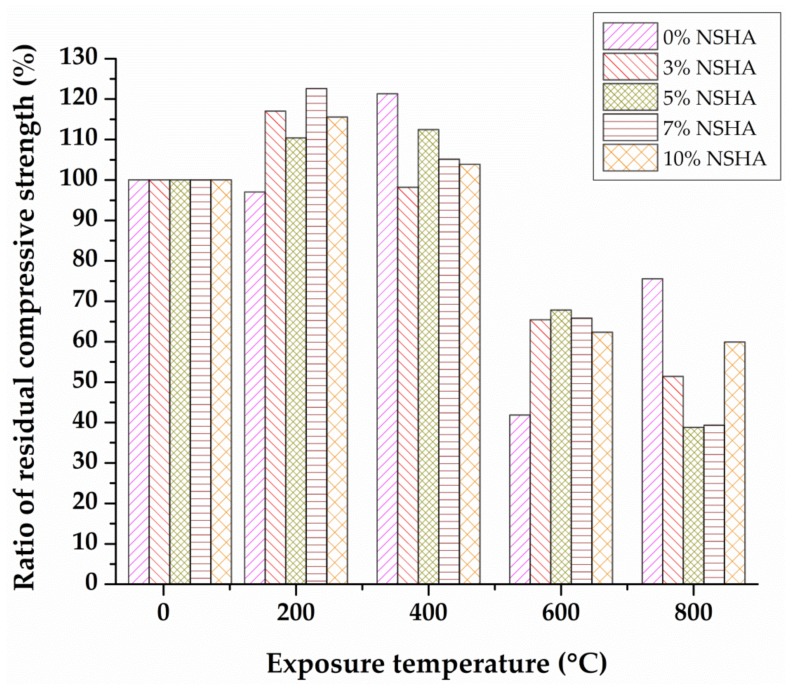
The ratio of residual compressive strength for the 7 days cured concrete.

The outstanding and reduced compressive strength for the heated concrete samples after 7 days curing period is demonstrated in Figure 7. Increasing the optimum amount of NSHA gradually raised the residual compressive strength more than for the control mix likely to be caused by the pozzolanic effect of NSHA during the hydration process, which leads to producing a high amount of C–S–H, which is responsible for strength development. According to the previous study [24] strength improved at 200 °C and it started decreasing at the 400 °C, 600 °C and 800 °C levels of temperature exposure. In this study, the concrete’s early age residual strength was observed to be superior as compared to control mixes by 22.6%, and 12.4% at 200 °C and 400 °C, respectively, followed by a noticeable strength drop of 32.2% and 61.2% at 600 °C and 800 °C, respectively, for the 5% NSHA optimal replacement. Moreover, while the application of rice husk ash as investigated by Wang et al. [22] retained up to 50% of strength at 800 °C, the current study observed about 39% of strength being retained.

**Figure 8:**
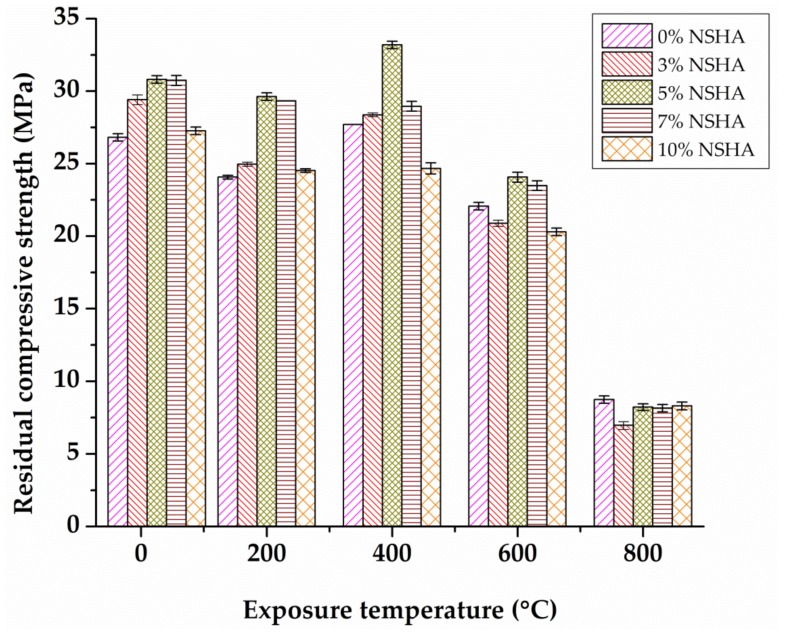
Residual compressive strength versus temperature for the 28 days cured concrete.

The residual strength for the 28 cured samples with their respective standard deviations is presented in Figure 8. A similar trend was observed for the concrete samples cured for 28 days for samples with 3%, 5% and 7% NSHA. The cement-NSHA blended concrete performed better than the control mix to high temperatures up to 400 °C, followed by a significant reduction in strength. 

**Figure 9:**
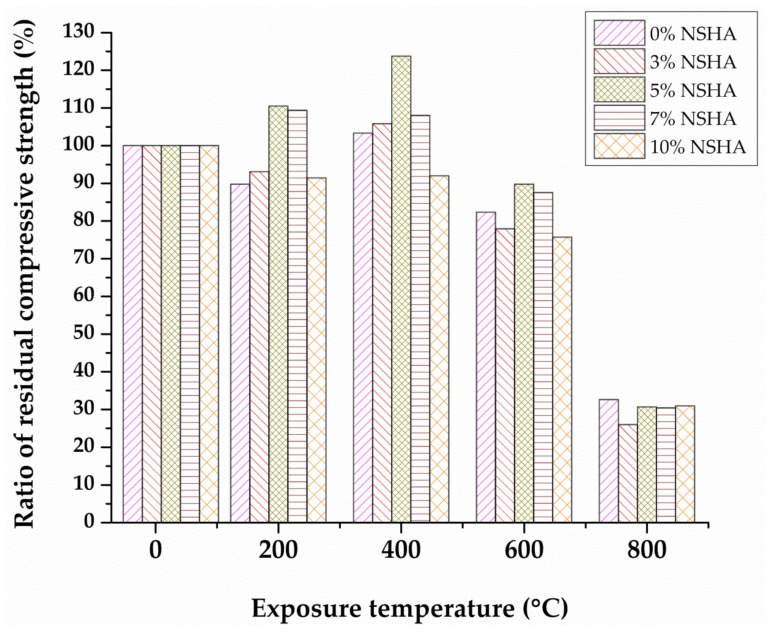
The ratio of residual compressive strength for the 28 days cured concrete.

Figure 9 shows the residual and decreased compressive strength for the 28 day cured concrete samples. The residual compressive strength of 5% NSHA concrete is 10.5% and 23.8% greater than the 0% NSHA concrete at 200 °C and 400 °C, respectively, and then it fell by 10.2% and 69.3% at 600 °C and 800 °C, respectively. Both NSHA and non-NSHA specimens show a drastic decrease in strength after exposure to 600 °C. However, the NSHA contained specimens that have a higher residual strength than the non-NSHA contained specimens. The higher strength of NSHA contained samples after subjection to high temperature is likely to be caused by the pozzolanic effect of NSHA during the hydration process, which leads to producing a high amount of C–S–H which is responsible for strength development. Additionally, NSHA acts like a micro filler and strengthens the microstructure of the system [40].

The observed early decrease of the compressive strength of concrete samples subjected to temperatures between 25 °C and 200 °C is due to the weakening of bonds likely to be ascribed by the swelling of water layers in the NHSA-cement paste [38]. The additional C–S–H phases produced by the pozzolanic reaction of NSHA with free lime that gets deposited in the pore system and the internal autoclaving where un-hydrated cement grains undergo further hydration, are responsible for the compressive strength rise as exposure temperature is raised. Undoubtedly, the results provide evidence that the partial replacement of OPC by 5% of NSHA escalates the compressive strength of cement concrete by about 24% higher than conventional control samples at 400 °C. Hence, the optimal replacement of cement by the synthetic pozzolanic material (NSHA) improves the cement paste’s capacity to resist fire up to 400 °C.

In general, it can be concluded that the reduction in strength of NSHA concrete is initiated by the dense microstructure, which caused the build-up of high internal pressure as a result of the water-vapor transition in the water interlayer. For temperatures ranging from 400 to 600 °C, a severe strength loss for all the types of concrete was observed and recorded as 17.7%, 22.1%, 10.2%, 12.4% and 24.6 % of the initial values for OPC, 0% NSHA, 3% NSHA, 5% NSHA, 7% NSHA and 10% NSHA concretes, respectively.

The cement paste contracts, and the aggregates expand upon exposing the concrete to high temperatures. Such a response leads to the weakening of the transition zone and bonding between aggregates and cement paste. This process, as well as the decomposition of the chemically formed hydration products, result in serious deterioration and strength loss in concrete exposed to elevated temperatures. An average strength loss of 70% was observed for concrete containing NSHA which deteriorated severely up to 800 °C due to the decomposition of C–S–H gel. Such an observation has been reported in previous studies [21,38]. 

Despite the improved early strength of concrete containing NSHA, a noticeable loss in compressive strength was observed at high temperatures as a result of built-up vapor pressure in a dense structure during the evaporation process of the physically and chemically bound water.

## 4. Conclusions

The present study examined the residual compressive strength and spalling effect of the cement-NSHA blended normal concrete (with characteristic strength of 25 MPa) subjected to elevated temperatures. The mixes containing NSHA exhibited greater compressive strength (33.2 MPa) than the control mix (26.7 MPa), implying that the inclusion of NSHA led to improved compressive strength both at ordinary and elevated temperatures. The blended concrete was found to perform better than the normal concrete when fired up to 400 °C. The strength decreased significantly for concrete specimens fired beyond 400 °C associated with higher mass losses due to spalling. The following deductions can be made based on the presented experimental results:

- For all concrete samples exposed up to 400 °C, there was no noticeable consequence on the surface. Significant fractures and localized spalling were noticed when the exposure temperature rose to 600 °C, and they were more pronounced at 800 °C. The color change was insignificant for all series of OPC and blended concrete considered.
- Specimens exposed to greater than 400 °C suffered spalling and mass loss. In previous findings by Bastami et al. [41], spalling was witnessed for specimens subjected to temperatures more than 300 °C. The spalling varied from slight aggregate spalling (characterized by surface pitting) to significant apportions of specimens being blown off with explosive force.
- The compressive strength of specimens fell notably for both normal concrete and NSHA blended concrete samples when exposed to the temperature exceeding 400 °C. The drop in compressive strength of about 60% and 69% was observed at 800 °C for the 7 and 28 days cured specimens, respectively.
- Grounded in the investigated strength and physical properties of NSHA concrete, it is evident that 5% NSHA concrete performed better than control concrete for all mixtures for temperatures up to 400 °C. This implies that the reaction of pozzolans with portlandite is favored between 100 °C and 400 °C, resulting in a considerable decrease of Ca(OH)_2_ content. Therefore, NSHA is worth being recommended as a pozzolanic material for structural applications in concrete structures, of which they will still perform at high temperatures up to 400 °C.

## Figures and Tables

**Table 1:** Measured properties of aggregates.

Properties	Coarse Aggregates	Fine Aggregates
Water absorption (%)	1.65	1.89
Specific gravity	3.12	2.77
Bulk density (kg/m^3^)	1465	1590
Moisture content (%)	3.48	4.99

**Table 2:** Concrete mix design.

Components (kg/m^3^)	Percent of OPC Replacement (wt%)				
	0	3	5	7	10
Cement	410	397.7	389.5	381.3	369
NSHA	0	12.3	20.5	28.7	41
Sand	530	530	530	530	530
Aggregates	1260	1260	1260	1260	1260
Water	205	205	205	205	205

**Table 3:** Chemical composition of cement and NSHA materials.

Chemical Properties	Cement (%)	NSHA (%)
SiO_2_	17.4	45.2
CaO	57.4	2.6
Al_2_O_3_	4.9	22.6
Fe_2_O_3_	3.0	3.4
MgO	0.5	3.3
SO_3_	2.7	0.8
Loss on Ignition	9.9	5.6
Blaine-specific surface area (m^2^/kg)	431	544
Initial setting time (minutes)	147	–
Compressive strength 28 days (MPa)	46	–
Water absorption (%)	–	1.5

**Table 4:** Physical characteristics of concrete mixes at elevated temperatures.

Mix	200 °C	400 °C	600 °C	800 °C
0% NSHA	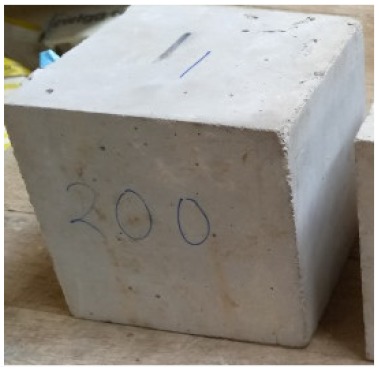	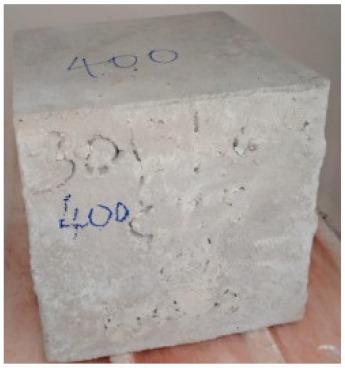	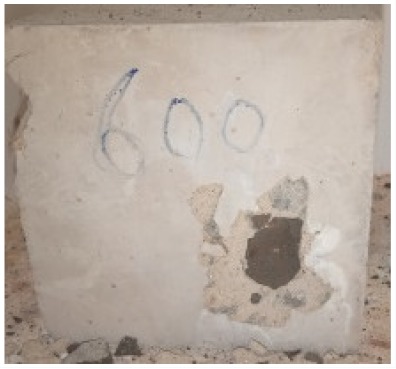	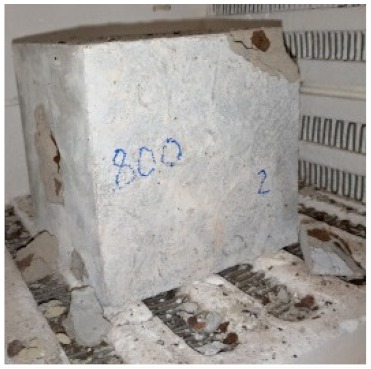
3% NSHA	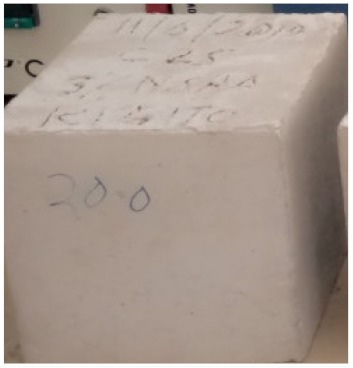	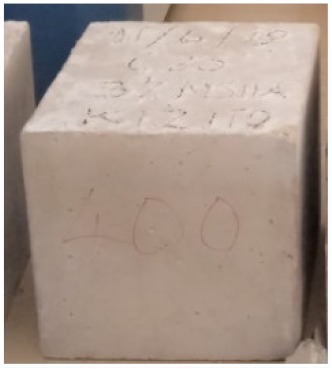	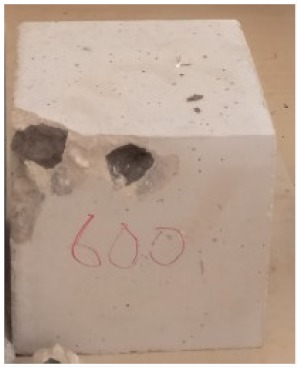	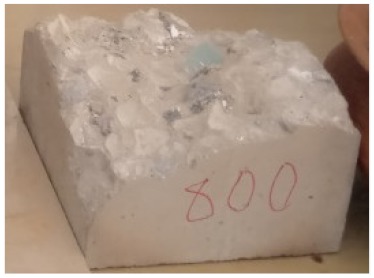
5% NSHA	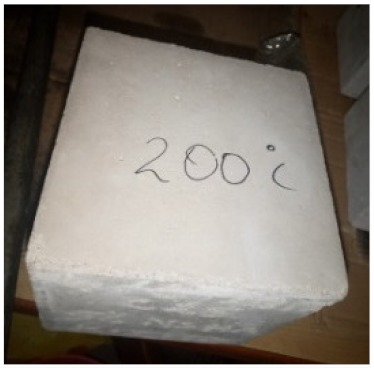	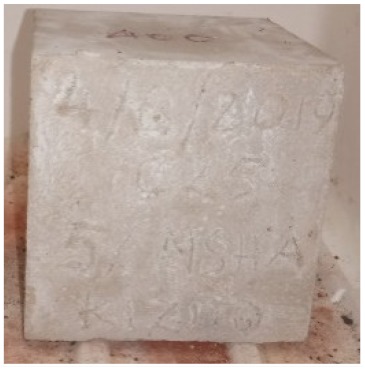	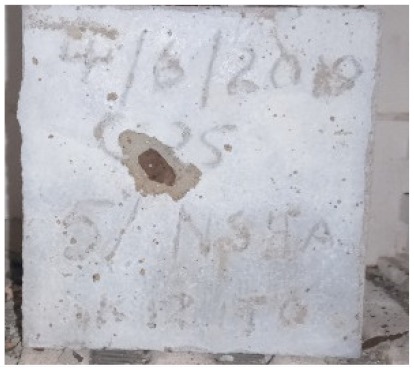	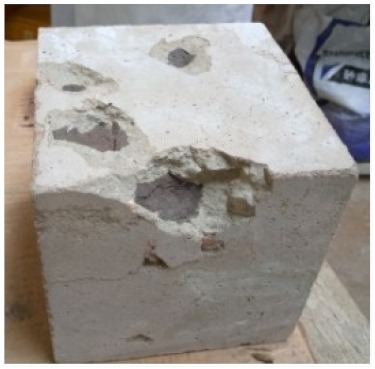
7% NSHA	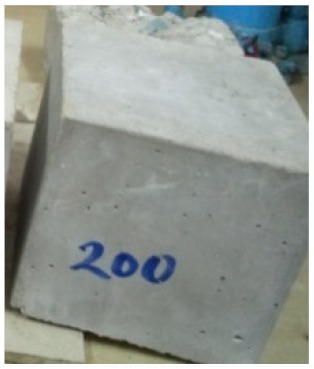	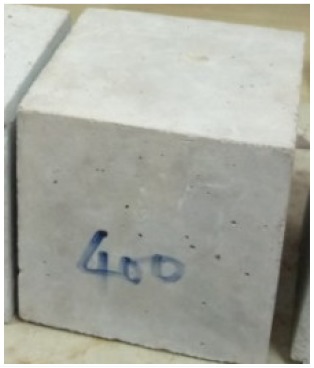	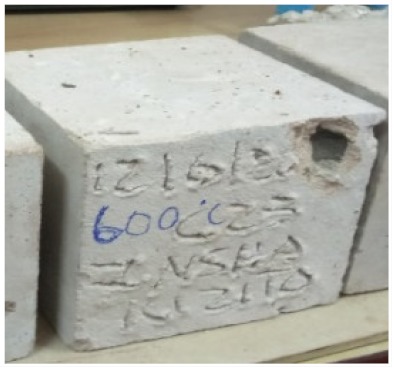	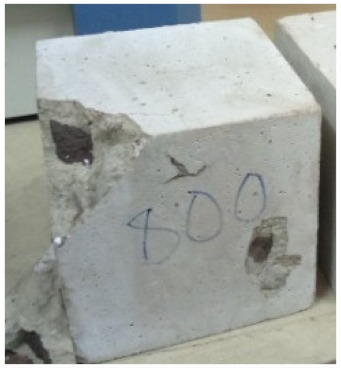
10% NSHA	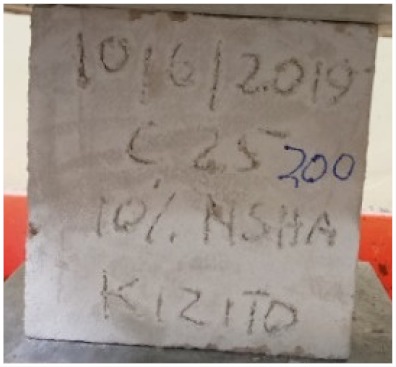	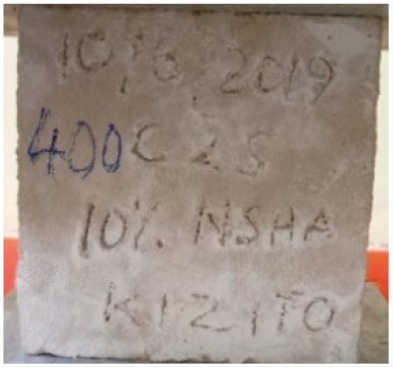	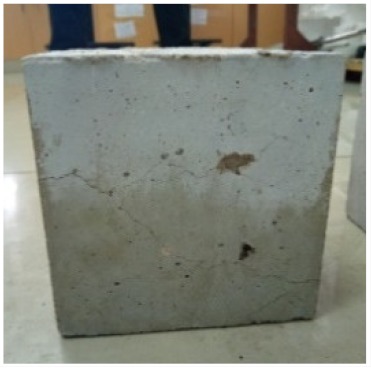	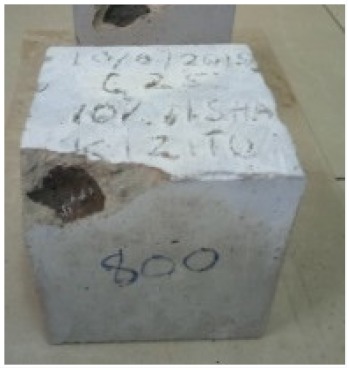

**Table 5:** Residual compressive strength versus temperature for the 7 and 28 days cured concrete.

Exposure Temperature (°C)	Compressive Strength (MPa)									
	0% NSHA		3% NSHA		5% NSHA		7% NSHA		10% NSHA	
	7 Days	28 Days	7 Days	28 Days	7 Days	28 Days	7 Days	28 Days	7 Days	28 Days
25	19.7	26.8	20.9	29.4	24.3	30.8	20.0	30.7	20.0	27.3
200	19.1	24.1	24.4	25.0	26.8	29.3	24.5	29.3	23.1	24.5
400	23.2	27.7	24.0	28.4	30.1	33.2	25.8	29.0	24.0	24.7
600	9.7	22.1	15.7	20.9	20.4	24.1	17.0	23.5	15.0	20.3
800	7.3	8.7	8.1	7.0	7.9	8.2	6.7	8.1	9.0	8.3
